# Larger Thyroid Volume and Adequate Iodine Nutrition in Chinese Schoolchildren: Local Normative Reference Values Compared with WHO/IGN

**DOI:** 10.1155/2016/8079704

**Published:** 2016-11-24

**Authors:** Zhe Mo, Xiaoming Lou, Guangming Mao, Zhifang Wang, Wenming Zhu, Zhijian Chen, Xiaofeng Wang

**Affiliations:** Department of Environment and Occupational Health, Zhejiang Provincial Center for Disease Prevention and Control, Hangzhou, Zhejiang, China

## Abstract

*Objective*. Thyroid volume measured by ultrasound to define goiter needs reliable local thyroid volume reference from iodine-sufficient populations. The aim of this study is to explore the reference interval for normal thyroid volume in schoolchildren aged 8–10 years from Zhejiang Province, China.* Methods*. A probability-proportionate-to-size sampling method was applied to select a representative sample of 1213 children aged 8–10 years in Zhejiang Province to detect the thyroid volume, salt iodine, and urine iodine.* Results*. Median urinary iodine concentration in involved schoolchildren was 178.30 (125.00) *μ*g l^−1^, with the percentage of samples less than 100 *μ*g l^−1^ as 12.69% and more than 300 *μ*g l^−1^ as 15.25%. Thyroid volume was significantly correlated with age and anthropometric measurements independently of each other. The 97th percentile of thyroid volume in our study was larger generally than the new international reference.* Conclusions*. The iodine nutritional status in Zhejiang Province was at an adequate level. Despite some limitations in this study, we initially established the reference values for thyroid volume in 8–10-year-old schoolchildren in Zhejiang Province, China, as a local reference to be used for monitoring iodine deficiency disorders.

## 1. Introduction

Goiter prevalence in school-age children is an important indicator of iodine deficiency disorders in a population [1]. Several studies [2–4] have shown that thyroid volume measured by ultrasound is the preferred indicator in epidemiological studies and monitoring of iodine deficiency disorders. Nevertheless, thyroid volume measured by ultrasound needs reliable reference intervals for normal thyroid volume from iodine-sufficient populations.

There are many controversial issues [5–13] about defining the reference intervals for normal thyroid volume. In 2004, Zimmermann et al. [13] established the international reference intervals for thyroid volume by ultrasound adjusted for age and body surface area (BSA). These were based on 3529 schoolchildren living in areas with long-standing iodine sufficiency; age and BSA-specific upper normal limits of thyroid volume were provided by sex. These reference intervals were adopted by the World Health Organization (WHO) and Iodine Global Network (IGN) as the recommendation of thyroid volume in 2007 [1] and suggested that the population-specific references for thyroid volume in countries with long-standing iodine sufficiency may be more accurate than a single international reference.

The Universal Salt Iodization program has been mandatory since 1994 in China; iodine nutrition indicated by median urinary iodine concentrations (MUIs) is of long-standing sufficiency [14, 15]. However, local endemic goiter diagnostic standards [16], which ignored the growth and development factor of children, are now based on the reference published in 1993 [5].

We previously performed studies [17, 18] on the reference intervals for normal thyroid volume in schoolchildren of Zhejiang Province, China. However, as noted, those studies ignored the growth and development factor of children and were based on a relatively small number of observations. Few recent data on urinary iodine and thyroid volume in Chinese schoolchildren are available. The objective of this study is to explore the reference interval for normal thyroid volumes in schoolchildren aged 8–10 years from Zhejiang Province and compare these with WHO/IGN recommended references.

## 2. Subjects and Methods

### 2.1. Subjects

**Figure 1:**
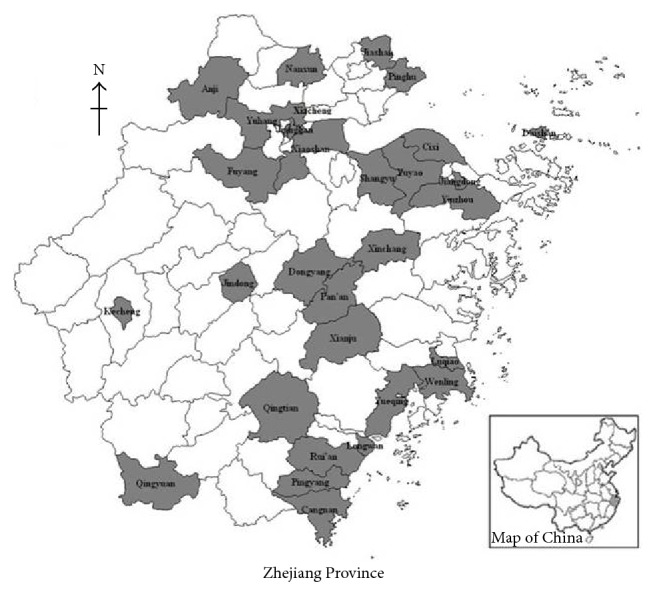
Sampling counties in Zhejiang Province, China. Thirty counties (gray areas) from Zhejiang Province, in the east coastal region of China, which covers 89 counties, were selected by using a probability-proportionate-to-size sampling method.

This study was conducted in Zhejiang Province, an eastern coastal region of China that covers 89 counties. In the study, 30 counties from Zhejiang Province (Figure 1) were selected by using a probability-proportionate-to-size (PPS) sampling method; the design used current census data to provide a systematic sampling of communities based on the cumulative population. Next cluster sampling was applied to select one primary school from each selected county, and schoolchildren aged 8–10 were randomly selected in each school as investigation subjects. Data were collected from April to September 2013.

The sample size of PPS, estimated by the *N* = *Z*_*α*_^2^ × goiter × (1 − goiter)×(Design effect)/*ε* ^2^ formula, assumed that *α* = 0.05, *Z*_*α*_ = 1.96, goiter = 5%, *ε* = 0.02, and design effect = 2.5. The sample size equaled (1.96)^2^ × 5% × 95% × 2.5/(0.02)^2^ = 1140, and because of loss to follow-up the investigation sample size was expanded to 1440. Hence, each school randomly selected 48 schoolchildren aged 8–10 (24 boys and 24 girls, with 16 groups consisting of one 8-year-old, one 9-year-old, and one 10-year-old, resp.) to be investigated.

### 2.2. General Survey

Subjects were questioned with parental guidance and introduced to the study protocol. The questionnaire involved demographic characteristics, including age, gender, family income level, and history of disease.

### 2.3. Anthropometrics

The measure of height and weight was performed using WHO standard methods [21] before measurement of thyroid volume, requiring the subjects to take off shoes and belt and wear light clothes. Height measurements were precise to 0.1 cm and weight to 0.1 kg. The formula of weight (kg)^0.425^ × height (cm)^0.725^ × 71.84 × 10^−4^ [22] was used to calculate BSA (m^2^).

### 2.4. Urinary Iodine

The instant urine samples of all subjects were collected in plastic tubes, placed at –4°C before being sent to the laboratory, and analyzed for urinary iodine concentrations (UICs) by spectrophotometer method (WS/T 107-2006) [23]. According to the recommended iodine nutrition status evaluation criteria of WHO [1], severe iodine deficiency was indicated by MUIs of schoolchildren <20 *μ*g l^−1^, moderate iodine deficiency by 20 *μ*g l^−1^ ≤ MUIs < 49 *μ*g l^−1^, mild iodine deficiency by 50 *μ*g l^−1^ ≤ MUIs < 99 *μ*g l^−1^, adequate iodine nutrition by 100 *μ*g l^−1^ ≤ MUIs < 199 *μ*g l^−1^, above requirements by 200 *μ*g l^−1^ ≤ MUIs < 299 *μ*g l^−1^, and excessive iodine by MUIs ≥ 300 *μ*g l^−1^.

### 2.5. Salt Iodine

A 10 g edible salt sample was collected from schoolchildren's homes, stored at room temperature, and analyzed for salt iodine concentrations by colorimetric titration method (GB/T 13025.7-2012) [24]. According to the national food safety standard-iodine content of edible salt (GB 26878-2011) [25], the acceptable range of iodine content in salt is 18–33 mg kg^−1^.

### 2.6. Thyroid Volume

Thyroid volume was examined by a single, experienced operator, using a portable ultrasound machine (PICO, SonoAce, Korea) with a 7.5-MHZ, 60 mm transducer. During the examination, subjects sat straight on the chair with their neck exposed, and the operator measured the maximum width, depth, and length of the thyroid lobe from the right to left lobe and detected thyroid nodules. The thyroid capsule was not included. Thyroid volume was the sum of right and left thyroid lobe volumes; the lobe volume equals width (cm) × depth (cm) × length (cm) × 0.479 [26].

### 2.7. Ethics

This study was approved by the ethics committee of the Zhejiang Provincial Centre for Disease Prevention and Control (CDC). Informed written consent was obtained from the parents or guardians of the children.

### 2.8. Statistical Analysis

Based on the result of survey and laboratory tests, counties in which MUIs were within the range of 100 to 299 *μ*g l^−1^ and in which schoolchildren had no history of thyroid disease, had no thyroid nodules, and used the qualified iodized salt were considered eligible for inclusion in our study.

Continuous variables such as age, height, weight, and BSA, which were normally distributed according to the Shapiro-Wilk test, were described as mean ± standard deviations (SD). UICs that were not normally distributed according to the Shapiro-Wilk test were presented as median (interquartile range [IQR]). Thyroid volume was expressed as geometric mean ± SD, because logarithmically transformed values of thyroid volume were normally distributed.

Differences in original and analysis data for sex and age were analyzed by Chi-square test and by Wilcoxon test for UICs. Differences in different age or sex groups for height, weight, BSA, and thyroid volume in log scales were analyzed by one-way analysis of variance (ANOVA) or two-sample* t*-test and by Wilcoxon test or Kruskal-Wallis test for UICs. Differences between UIC groups for thyroid volume in log scales were analyzed by ANOVA.

Spearman correlation coefficient was calculated to reflect the correlation of UICs with height, weight, BSA, and thyroid volume in log scale, and the Pearson correlation coefficient was used to reflect the correlation on BSA with height and weight. Variance inflation factor was calculated to identify collinearity in the independent variables. Univariate and multivariate linear regression analyses were applied to identify the factor effect on thyroid volume in log scale. The 97th percentile (P97) of thyroid volume was calculated to compare with WHO/IGN recommended references and other national references.

All significance tests were two-tailed, and the level of significance was *p* < 0.05. Data processing and statistical calculations were performed by using SAS 9.3 software (Cary, NC, USA).

## 3. Results

### 3.1. General Survey

A total of 1549 schoolchildren from 30 counties in Zhejiang Province were investigated and examined. MUIs were 178.40 (127.05) *μ*g l^−1^, and MUIs of 30 counties were within the range of 116.50 (38.00) *μ*g l^−1^ to 285.00 (157.00) *μ*g l^−1^. Hence, all counties were included in our study. According to the inclusion criteria, schoolchildren who had a history of thyroid disease (total 1: 1 boy and 0 girls) or thyroid nodules (total 102: 38 boys and 64 girls) or did not use the qualified iodized salt (total 162: 93 boys and 69 girls) or did not submit complete information (total 71: 39 boys and 32 girls) were excluded.

Finally, a total of 1213 schoolchildren were included in this study. These represented about 9/10,000 children in 8–10-year-old groups in Zhejiang Province [27]. There were no statistically significant differences between original data and analysis data in sex and age (*χ* ^2^_sex_ = 0.005; *χ* ^2^_age_ = 0.16; all *p* > 0.05). The sample included 610 boys and 603 girls, of which 380 were 8-year-olds, 419 were 9-year-olds, and 414 were 10-year-olds. On the whole, the height of all involved schoolchildren was 134.58 ± 7.65 cm, weight was 30.48 ± 6.70 kg, BSA was 1.07 ± 0.13 m^2^, and thyroid volume was 3.49 ± 1.30 ml. Meanwhile, height, weight, BSA, and thyroid volume in log scale increased with an increase of age (*F*_height_ = 265.05, *F*_weight_ = 107.63, *F*_BSA_ = 176.19, *F*_thyroid volume_ = 101.94, and all *p* < 0.05). Boys had higher weight and BSA than girls (*t*_weight_ = 12.83, *t*_BSA_ = 8.74, and all *p* < 0.05), but no differences by sex for height and thyroid volume in log scale were observed (*t*_height_ = 0.74, *t*_thyroid volume_ = 2.25, and all *p* > 0.05). See Table 1.

### 3.2. Urinary Iodine Concentrations

 MUI in the involved schoolchildren was 178.30 (125.00) *μ*g l^−1^, with the percentage of samples less than 100 *μ*g l^−1^ as 12.69% and more than 300 *μ*g l^−1^ as 15.25%. MUIs in 30 counties were within the range of 115.50 (39.00) *μ*g l^−1^ to 278.00 (121.00) *μ*g l^−1^. There were no statistically significant differences between original data and analysis data for UICs (*Z* = 0.02; *p* > 0.05). Boys had higher levels of UICs than girls (*Z* = 2.09; *p* < 0.05), but differences in age for UICs were not observed (*χ* ^2^ = 0.46; *p* > 0.05). Meanwhile, there was no significant correlation in UICs with height, weight, BSA, and thyroid volume in log scale (*r* = 0.01, 0.49, 0.51, and 0.14; all *p* > 0.05). See Table S1 in Supplementary Material available online at http://dx.doi.org/10.1155/2016/8079704.

### 3.3. Factors regarding Thyroid Volume

From the result of univariate linear regression analysis, thyroid volume in log scale was significantly associated with age, height, weight, and BSA but not with sex and UICs which was presented in Table 2.

BSA was a composite indicator calculated by height and weight. There were strong correlations in BSA with height and weight (*r* = 0.86 and 0.97; all *p* < 0.05), and collinearity was considered in multivariate analysis (variance inflation factor = 1.98). Hence, height and weight were excluded from multivariate analysis.

From the result of multivariate linear regression analysis (Table 3), only age and BSA independently were found to have a significant effect on thyroid volume in log scale after being adjusted for sex and UICs; the adjustment *R* ^2^ was 0.39.

Geometric mean of thyroid volume increased with an increase of UICs with significant differences in the eight-year-old groups (*F* = 3.59; *p* < 0.05), and there was a distinct U-shaped curve relationship between the geometric mean of thyroid volume and UICs at BSA of 1.2 m^2^ (*F* = 4.14; *p* < 0.05), which is presented in Tables 4 and S2.

### 3.4. Thyroid Volume

The results in Tables S3 and S4 show that age-specific P97 of thyroid volume in boys' and girls' groups averaged 32.41% (28.13%–34.88%) and 22.68% (19.68%–24.47%) higher, respectively, than that in the 2007 WHO/IGN recommended reference. On the other hand, BSA-specific P97 of thyroid volume in boys' and girls' groups, except the groups in which the number observed was less than 30, also averaged 38.64% (28.10%–45.31%) and 25.81% (19.68%–48.80%), higher than those in the 2007 WHO/IGN recommended reference.

### 3.5. Compare with Other References

**Figure 2:**
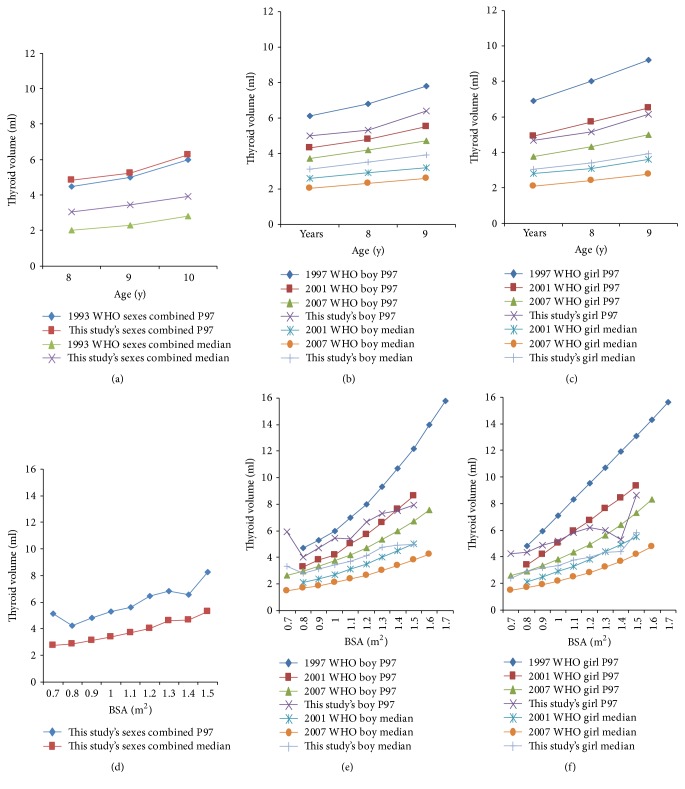
Comparison of median and P97 of thyroid volume between this study and WHO/IGN recommended references by age and BSA. (a), (b), and (c) show reports published from 1993 to 2007 [1, 6, 19, 20]; (d), (e), and (f) show reports published from 1997 to 2007 [1, 6, 20].

The age or BSA-specific median and P97 of thyroid volume by sex in our study were compared with the WHO/IGN recommended references data (Figure 2).  Figure 2(a) showed that our age-specific P97 of thyroid volume in sexes combined data was very similar and nearly identical to the values found in 1993 but those in median were slightly higher than the values found in 1993. Figures 2(b) and 2(c) show that age-specific median and P97 of thyroid volume in boys' and girls' groups were nearly similar to the values found in 2001.

Figures 2(e) and 2(f) showed that our BSA-specific median and P97 of thyroid volume in boys' and girls' groups were similar to the values found in 2001 but were higher than those that were published in 2007. Overall, our P97 of thyroid volume was approximately 20–30% smaller than that found in 1997, irrespective of whether thyroid volume was expressed as a function of age or BSA.

The comparison of this study to the data from other national references is presented in Figures S1 and S2. Our age-specific median and P97 of thyroid volume data with sexes combined were very similar to data from United States and Iran but were about 44% in median and 20% in P97 higher than those from Japan. Our age-specific P97 of thyroid volume in boys' and girls' groups was similar to data from Malaysia and Brazil but higher than that from Japan and, meanwhile, smaller than that from Turkey; in girls it was similar to that from Poland, but in boys, it was smaller than that from Poland. However, it had different patterns in median of thyroid volume data. Our age-specific median of thyroid volume in boys' and girls' groups was higher than that from Malaysia and Japan, and in boys it was similar to that from Brazil, Turkey, and Poland, but in girls, it was smaller than that from Brazil, Turkey, and Poland. Our BSA-specific median and P97 of thyroid volume data with sexes combined were, on average, about 20%–40% in median and 20%–30% in P97 higher than those from United States and Japan. Our BSA-specific median and P97 of thyroid volume in the boys' and girls' groups were similar to data from Malaysia, higher than Japan, and lower than those in Italy.

**Figure 3:**
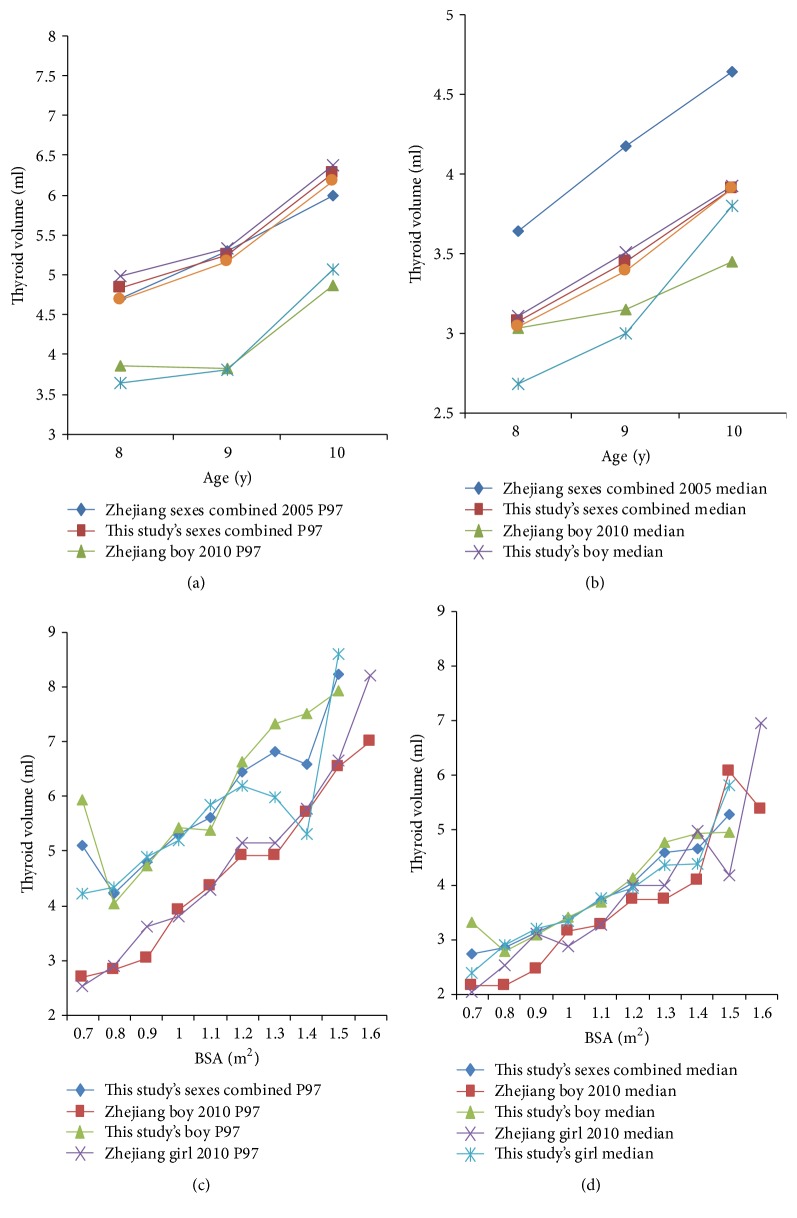
Comparison of median and P97 of thyroid volume between this study and previous reports in Zhejiang Province, China, by age and BSA. (a) and (b) show studies carried out from 2005 to 2013 [17, 18]; (c) and (d) show studies carried out from 2010 to 2013 [18].

Comparisons of previous reports about thyroid volume in Zhejiang Province are shown in Figure 3. Our age-specific P97 of thyroid volume data with sexes combined was very similar to the data of Zhejiang in 2005 but higher than the data from boys and girls of Zhejiang in 2010, respectively. However, it had different patterns in median of thyroid volume data. Our age-specific median of thyroid volume data with sexes combined was lower than the data of Zhejiang in 2005 but higher than the data from boys and girls of Zhejiang in 2010, respectively. Meanwhile, our BSA-specific median and P97 of thyroid volume in boys' and girls' groups were also higher than the data of Zhejiang in 2010.

## 4. Discussion

In this study, the MUI in the study's schoolchildren was 178.30 *μ*g l^−1^, which was similar to the data reported in Germany (183.00 *μ*g l^−1^) [28] and Bahrain (178.00 *μ*g l^−1^) [13] but higher than that in Turkey (53.00 *μ*g l^−1^) [29], Bangladesh (73 *μ*g l^−1^) [10], Switzerland (115 and 118.00 *μ*g l^−1^) [11, 13], Sweden (125.00 *μ*g l^−1^) [30], Italy (125.00 *μ*g l^−1^) [31], Malaysia (132.80 *μ*g l^−1^) [9], Netherlands (154.40 *μ*g l^−1^) [32], and Poland (126.60–155.10 *μ*g l^−1^) [33] and less than that in South Africa (191.00 *μ*g l^−1^) [13], Iran (212.00 *μ*g l^−1^) [34], Peru (253.00 *μ*g l^−1^) [13], Philippines (279.00 *μ*g l^−1^) [35], the United States (282.00 and 285.00 *μ*g l^−1^) [10, 13], Japan (281.60 and 288 *μ*g l^−1^) [13, 36], and Brazil (360.00 *μ*g l^−1^) [37]. Compliance with the Universal Salt Iodization program has been mandatory since 1994 in Zhejiang Province, China, which was identified as iodine sufficient for decades in several sustained surveys from 1995 to 2011 [38]. In our study, MUIs of all 30 counties were within the range of 100–300 *μ*g l^−1^, and the percentage of total samples, which was less than 50 *μ*g l^−1^, was less than 5%. Thus, most schoolchildren in our study population have spent their entire life in long-standing iodine-sufficient areas. Meanwhile, it is important to include subjects considered normal for reference, so those schoolchildren who had a history of thyroid disease or thyroid nodules or did not use the qualified iodized salt or did not submit complete information were excluded before the data analysis. Overall, we feel confident that our sample was from long-standing iodine-sufficient areas and included normal subjects.

It is well known that thyroid volume could be affected by genetic features in growth and development as well as environmental factors, including different iodine dietary intakes [13, 31, 36]. MUIs in our samples indicated iodine sufficiency, so genetic features in growth and development may have mainly contributed to the influence on thyroid volume. In our study, as in others [30, 31, 35, 36, 39], BSA and age, independently of each other, significantly positively influenced thyroid volume; these parameters are used to assess thyroid volume as adjusted predictors. However, regarding the correlation between thyroid volume and sex, UICs were not obtained, and these findings were in line with the results reported in most iodine-sufficient areas [10, 36, 37]. Interestingly, in thyroid volume, no differences in BSA and weight have been found between boys and girls, but significant difference in height suggested that height might not be an important predictor of thyroid volume compared to weight and BSA. Moreover, because only a 31% variation of thyroid volume can be determined by age and BSA on the multivariate linear regression, the major variation of thyroid volume could be explained by other factors, such as race, and significant differences in thyroid volume exist between several countries [13]. Therefore, the new international reference supports the use of a single, site-independent set of references in different countries [13]. Until now, the local thyroid volume reference data had been established in many countries [9, 11, 30, 32, 33, 36, 37]. Thus, we conducted this study to explore the references in our province.

The P97 of thyroid volume in our study of both sexes was higher than the new international reference in 2007 [1], irrespective of whether it was expressed as a function of age and BSA, and similar to the reference in 1993 [19] and 2001 [20] but lower than the reference in 1997 [6], when the data had a systematic measures bias that resulted in higher values, which were corrected to issue the reference in 2001 [20]. The larger thyroid volume was probably not determined by lower MUIs than those (MUIs: 203 *μ*g l^−1^) [13] in the new international reference population because there is no overall correlation between thyroid volume and UICs during times of iodine sufficiency [13, 18], which was consistent with the results of our study, as mentioned earlier, although with a slight difference between the two at 8 years of age and 1.2 m^2^ of BSA.

Two earlier studies reported thyroid volume in schoolchildren of Zhejiang Province [17, 18], which provided earlier data for this study, although there were some limitations in those studies. Compared with the data from the study in 2005 [17], the age-specific P97 of thyroid volume in our study was very similar to those reference values, indicating the reproducibility of the results. Unfortunately, the growth and development factors of children were not measured in the study of 2005, so the BSA-specific P97 could not be presented. The data from the study in 2010 [18] was applied by polynomial regression to fit the data, but this study was based on a relatively small number of observations with a larger age range.

In the comparison of thyroid volume in different countries, as in the results of Zimmermann et al. [13], there were significant differences between countries in age and BSA-adjusted P97 of thyroid volume. At the age-specific P97, our values were similar to the data reported in Malaysia [9], the United States [10], Iran [34], and Poland [33] in girls from iodine-sufficient areas and the data reported in Brazil [37] from iodine-excessive areas but higher than those in Japan [36] from iodine-sufficient areas and lower than those in Turkey [29] and Poland [33] in boys from iodine-sufficient areas. At the BSA-specific P97, our values were similar to the data reported in Malaysia [9] but higher than those in Japan [36] and United States [10] and lower than those in Italy [31] from iodine-sufficient areas. Overall, the P97 of thyroid volume in our study by age and BSA was similar to those in Malaysia [9], which is located in Southeast Asia near the southern area of China, and it had almost the same age composition as our two samples. However, our P97 values were far higher than those in Japan [36], near the northeast area of China, which is a special one with small thyroid gland volume and high iodine excretion. Meanwhile, our P97 values were lower than those in European counties such as Turkey [29], whereas iodine deficiency control programs have not been successful up to recent years, and the higher values may reflect moderate iodine deficiency (MUIs: 53 *μ*g l^−1^), and as in Poland [33] and Italy [31], whereas these differences could be attributed to the differences in body size or ethnicity in these samples.

There were some limitations in this study. First, precision was not estimated by examining thyroid volume of the same subjects twice, although thyroid volume in our study was examined by a single experienced operator. Second, it is difficult to calculate the interobserver variation with the new international reference of 2007 [1], which could contribute partially to the difference between two samples. Third, the sample size of some BSA-specific groups of children, especially for 0.7, 1.4, and 1.5 m^2^ groups, is not enough to make final decision.

## 5. Conclusions

Our data suggested that the iodine nutritional status in Zhejiang Province was at an adequate level. Thyroid volume in our study shows a significant correlation with age and anthropometric measurements, independent of each other. The P97 of thyroid volume in our study was larger generally than the new international reference and similar to the previous WHO/IGN reference, which on the one hand appears acceptable for local use. Because of the limitations mentioned earlier, another study should be performed to complete the regional reference.

## Supplementary Material

Comparison on median and P97 of thyroid volume between this study and other reports by age and BSA were available in Supplementary Figure S1 and S2, Table S3 and S4. Urinary iodine concentrations and comparison of Geometric mean of thyroid volume according to group of UICs by BSA were available in Supplementary Table S1 and S2.

## Figures and Tables

**Table 1:** Descriptive statistics of schoolchildren participating in study.

Age (years)	Sex	*N*	Height (cm) ($\overset{-}{x}\pm SD$)	Weight (kg) ($\overset{-}{x}\pm SD$)	BSA (m^2^) ($\overset{-}{x}\pm SD$)	Thyroid volume (ml) (${\overset{-}{x}}_{Geometric}\pm SD$)
8	Boy	191	129.58 ± 6.46	28.32 ± 5.54	1.01 ± 0.11	3.13 ± 1.28
8	Girl	189	128.50 ± 5.77	26.18 ± 4.52	0.97 ± 0.09	3.03 ± 1.26
9	Boy	206	135.25 ± 6.00	30.89 ± 5.98	1.08 ± 0.11	3.53 ± 1.25
9	Girl	213	134.27 ± 6.43	29.60 ± 5.91	1.05 ± 0.11	3.39 ± 1.26
10	Boy	213	138.95 ± 6.87	33.96 ± 7.67	1.15 ± 0.14	3.90 ± 1.30
10	Girl	201	140.05 ± 6.60	33.38 ± 6.60	1.14 ± 0.12	3.90 ± 1.27
8		380	129.04 ± 6.14	27.25 ± 5.16	0.99 ± 0.10	3.06 ± 1.27
9		419	134.75 ± 6.23	30.23 ± 5.97	1.07 ± 0.11	3.46 ± 1.25
10		414	139.48 ± 6.75	33.68 ± 7.17	1.15 ± 0.13	3.90 ± 1.28
	Boy	610	134.77 ± 7.49	31.16 ± 6.88	1.08 ± 0.13	3.53 ± 1.30
	Girl	603	134.39 ± 7.81	29.79 ± 6.45	1.06 ± 0.13	3.42 ± 1.28

Total		1213	134.58 ± 7.65	30.48 ± 6.70	1.07 ± 0.13	3.49 ± 1.30

SD: interquartile range.

**Table 2:** Univariate regression for thyroid volume in log scale as a function of sex, age, height, weight, BSA, and UICs.

Variable	*β* (95% CI)	Standardized estimate	Standard error	*t*	*p*
Sex	0.02 (–0.01–0.05)	0.04	0.01	1.50	0.1335
Age	0.12 (0.10–0.14)	0.38	0.01	14.28	<0.0001
Height	0.01 (0.01–0.02)	0.42	0.00	16.07	<0.0001
Weight	0.02 (0.02–0.02)	0.44	0.00	17.11	<0.0001
BSA	0.91 (0.81–1.00)	0.47	0.05	18.30	<0.0001
UICs	0.000024 (–0.000007–0.000055)	0.04	0.00	1.49	0.1373

BSA: body surface area; UICs: urinary iodine concentrations; CI: confidence limits.

**Table 3:** Multivariate regression for thyroid volume in log scale as a function of sex, age, BSA, and UICs.

Variable	*β* (95% CI)	Standardized estimate	Standard error	*t*	*p*
Intercept	−0.12 (−0.27–0.03)	0.00	0.07	−1.59	0.1113
Sex	0.01 (−0.02–0.03)	0.01	0.01	0.43	0.6657
Age	0.07 (0.05–0.08)	0.20	0.01	7.32	<0.0001
BSA	0.72 (0.60–0.82)	0.37	0.04	12.84	<0.0001
UICs	0.00002349 (−0.00000356–0.00005054)	0.00	0.04	1.70	0.0887

BSA: body surface area; UICs: urinary iodine concentrations; CI: confidence limits.

**Table 4:** Comparison of geometric means of thyroid volume according to group of UICs by age.

Age (years)	UICs (*μ*g l^−1^)							
	0–99		100–199		200–299		300–	
	*N*	${\overset{-}{x}}_{Geometric}\pm SD$	*N*	${\overset{-}{x}}_{Geometric}\pm SD$	*N*	${\overset{-}{x}}_{Geometric}\pm SD$	*N*	${\overset{-}{x}}_{Geometric}\pm SD$
8	47	2.89 ± 1.26	178	3.04 ± 1.26	97	3.08 ± 1.28	58	3.34 ± 1.28
9	45	3.41 ± 1.23	198	3.45 ± 1.24	119	3.41 ± 1.27	57	3.54 ± 1.25
10	62	4.05 ± 1.30	185	3.86 ± 1.28	97	3.91 ± 1.28	70	3.96 ± 1.30

UICs: urinary iodine concentrations; SD: interquartile range.
