# Supplementary material for: Larger Thyroid Volume and Adequate Iodine Nutrition in Chinese Schoolchildren: Local Normative Reference Values Compared with WHO/IGN
Source: Int J Endocrinol. 2016 Nov 24;2016:8079704. doi: 10.1155/2016/8079704 (PMC5143740; doi:10.1155/2016/8079704)
Supplement: Supplementary file 1 — Comparison on median and P97 of thyroid volume between this study and other reports by age and BSA were available in Supplementary Figure S1 and S2, Table S3 and S4. Urinary iodine concentrations and comparison of Geometric mean of thyroid volume according to group of UICs by BSA were available in Supplementary Table S1 and S2. [file 8079704.f1.docx]

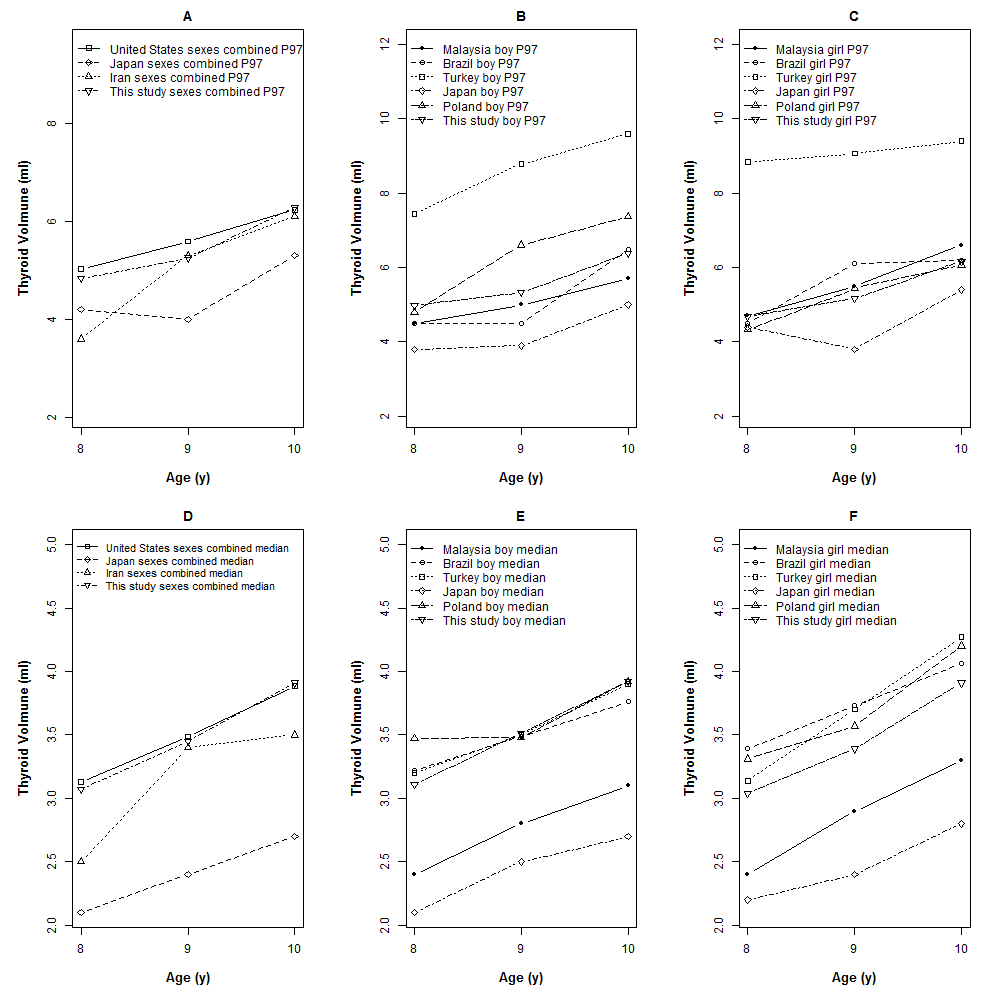


Figure S1: Comparison on median and *P97* of thyroid volume between this study and reports in different countries by age. The references from Malaysia, Poland and this study were based the data with adequate iodine nutrition, from Turkey with iodine deficiency, from United States, Japan and Iran with iodine above requirement, and from Brazil with iodine excessive, the order of horizontal axis was followed as reports published time from 1999 to 2012[[1-7](#_ENREF_1)]


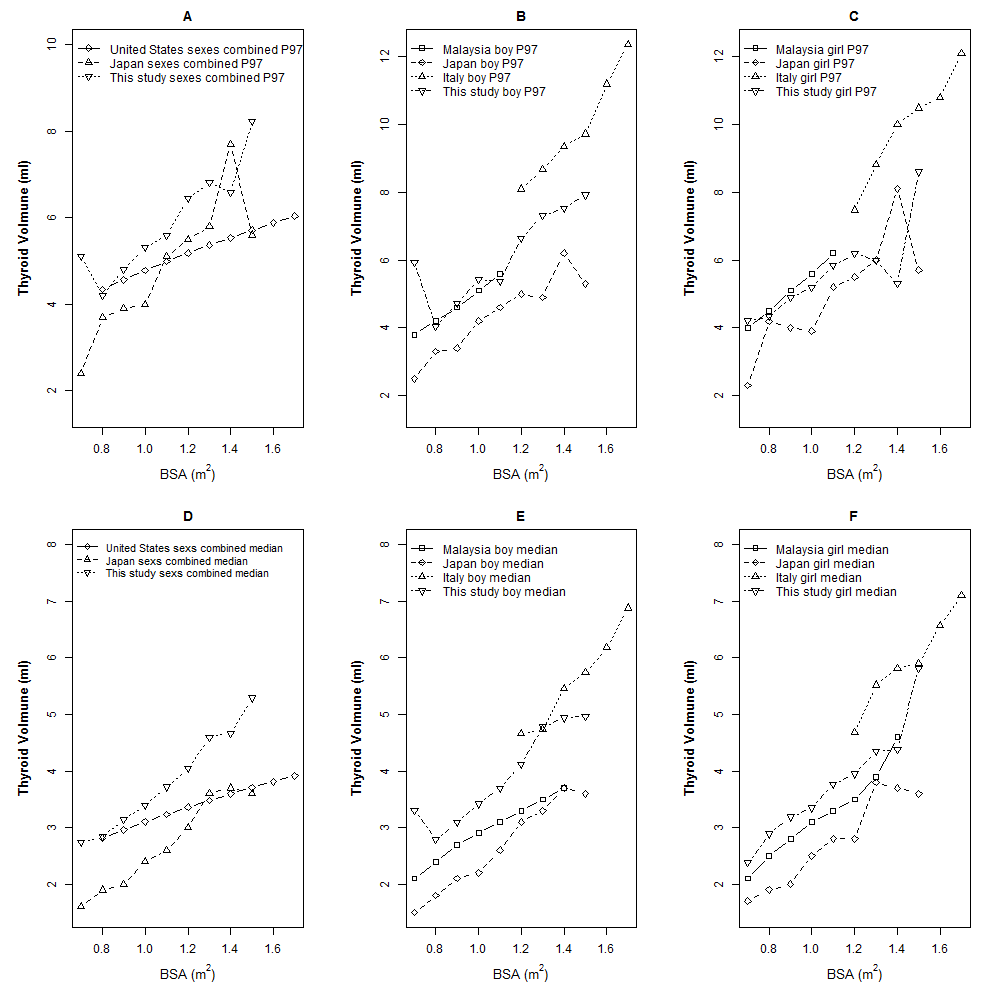


Figure S2: Comparison on median and *P97* of thyroid volume between this study and reports in different countries by BSA. The references from Malaysia, Italy and this study were based the data with adequate iodine nutrition, from United States and Japan with iodine above requirement, the order of legend was followed as reports published time from 1999 to 2009[[1](#_ENREF_1), [2](#_ENREF_2), [6](#_ENREF_6), [8](#_ENREF_8)]

Table S1: Urinary iodine concentrations in study

| Age(years) | Sex | N | MUIs (IQR) (µg l^-1^) | UICs frequency distribution (%) | | | | | |
| --- | --- | --- | --- | --- | --- | --- | --- | --- | --- |
|  |  |  |  | 0- | 20- | 50- | 100- | 200- | 300- |
| 8 | boy | 191 | 181.40(119.20) | 0.52 | 0.00 | 8.90 | 49.74 | 26.18 | 14.66 |
| 8 | girl | 189 | 171.83(120.12) | 0.53 | 3.17 | 11.64 | 43.92 | 24.87 | 15.87 |
| 9 | boy | 206 | 191.60(118.22) | 0.00 | 1.94 | 5.83 | 45.63 | 31.55 | 15.05 |
| 9 | girl | 213 | 174.90(104.00) | 0.00 | 1.41 | 12.21 | 48.83 | 25.35 | 12.21 |
| 10 | boy | 213 | 179.85(147.50) | 0.94 | 2.35 | 12.21 | 43.19 | 22.54 | 18.78 |
| 10 | girl | 201 | 169.00(124.70) | 1.00 | 1.00 | 12.44 | 46.27 | 24.38 | 14.93 |
| 8 |  | 380 | 177.75(124.95) | 0.53 | 1.58 | 10.26 | 46.84 | 25.53 | 15.26 |
| 9 |  | 419 | 181.64(116.00) | 0.00 | 1.67 | 9.07 | 47.26 | 28.40 | 13.60 |
| 10 |  | 414 | 175.00(139.00) | 0.97 | 1.69 | 12.32 | 44.69 | 23.43 | 16.91 |
|  | boy | 610 | 184.00(127.90) | 0.49 | 1.48 | 9.02 | 46.07 | 26.72 | 16.23 |
|  | girl | 603 | 171.80(117.20) | 0.50 | 1.82 | 12.11 | 46.43 | 24.88 | 14.26 |
| Total | | 1213 | 178.30(125.00) | 0.49 | 1.65 | 10.55 | 46.25 | 25.80 | 15.25 |

MUIs: median urinary iodine concentrations; IQR: inter-quartile range; UICs: Urinary iodine concentrations

Table S2: Comparison of Geometric mean of thyroid volume according to group of UICs by BSA

| BSA  (m^2^) | UICs (µg l^-1^) | | | | | | | |
| --- | --- | --- | --- | --- | --- | --- | --- | --- |
|  | 0-99 | | 100-199 | | 200-299 | | 300- | |
|  | N | $\overline{x}$_Geometric_ ±S.D | N | $\overline{x}$_Geometric_ ±S.D | N | $\overline{x}$_Geometric_ ±S.D | N | $\overline{x}$_Geometric_ ±S.D |
| 0.7 | 0 |  | 3 | 3.31±1.36 | 3 | 2.42±1.45 | 1 | 2.26±0.00 |
| 0.8 | 10 | 2.78±1.28 | 37 | 2.86±1.23 | 27 | 2.94±1.23 | 10 | 2.66±1.20 |
| 0.9 | 36 | 3.14±1.26 | 153 | 3.14±1.26 | 69 | 3.09±1.25 | 36 | 3.27±1.23 |
| 1.0 | 51 | 3.35±1.27 | 166 | 3.33±1.26 | 100 | 3.41±1.29 | 61 | 3.57±1.25 |
| 1.1 | 34 | 3.47±1.23 | 127 | 3.71±1.23 | 61 | 3.92±1.25 | 39 | 3.69±1.24 |
| 1.2 | 15 | 4.55±1.31 | 44 | 4.04±1.23 | 41 | 3.69±1.27 | 22 | 4.40±1.31 |
| 1.3 | 5 | 5.39±1.12 | 20 | 4.51±1.22 | 7 | 4.70±1.29 | 12 | 4.40±1.24 |
| 1.4 | 1 | 5.06±0.00 | 6 | 4.99±1.15 | 4 | 4.06±1.29 | 2 | 4.89±1.00 |
| 1.5 | 2 | 6.03±1.44 | 5 | 5.18±1.28 | 1 | 6.08±0.00 | 2 | 4.55±1.13 |

BSA: body surface area; UICs: Urinary iodine concentrations; S.D: inter-quartile range

Table S3: Compared median and P97 of thyroid volume in Chinese school children to 2007 WHO/ICCIDD[[9](#_ENREF_9)] recommend reference according to sex and age

| Age  (years) | Total | | | Boy | | | | | Girl | | | | |
| --- | --- | --- | --- | --- | --- | --- | --- | --- | --- | --- | --- | --- | --- |
|  | N | Median | *P97* | N | Median | *P97* | Median ^*^ | *P97* ^*^ | N | Median | *P97* | Median ^*^ | *P97* ^*^ |
| 8 | 380 | 3.07 | 4.83 | 191 | 3.11 | 4.98 | 2.03 | 3.71 | 189 | 3.04 | 4.68 | 2.08 | 3.76 |
| 9 | 419 | 3.45 | 5.25 | 206 | 3.51 | 5.33 | 2.30 | 4.19 | 213 | 3.39 | 5.17 | 2.40 | 4.32 |
| 10 | 414 | 3.91 | 6.28 | 213 | 3.92 | 6.38 | 2.59 | 4.73 | 201 | 3.91 | 6.17 | 2.76 | 4.98 |

^*^ Compared by 2007 WHO/ICCIDD recommend reference

Table S4: Compared median and P97 of thyroid volume in Chinese school children to 2007 WHO/ICCIDD[[9](#_ENREF_9)] recommend reference according to sex and BSA

| BSA (m^2^) | Total | | | Boy | | | | | Girl | | | | |
| --- | --- | --- | --- | --- | --- | --- | --- | --- | --- | --- | --- | --- | --- |
|  | N | Median | *P97* | N | Median | *P97* | Median ^*^ | *P97* ^*^ | N | Median | *P97* | Median ^*^ | *P97* ^*^ |
| 0.7 | 7 | 2.74 | 5.11 | 3 | 3.31 | 5.94 | 1.47 | 2.62 | 4 | 2.38 | 4.21 | 1.46 | 2.56 |
| 0.8 | 84 | 2.85 | 4.21 | 33 | 2.79 | 4.04 | 1.66 | 2.95 | 51 | 2.89 | 4.33 | 1.67 | 2.91 |
| 0.9 | 294 | 3.14 | 4.81 | 137 | 3.09 | 4.73 | 1.86 | 3.32 | 157 | 3.19 | 4.89 | 1.90 | 3.32 |
| 1.0 | 378 | 3.39 | 5.31 | 195 | 3.42 | 5.42 | 2.10 | 3.73 | 183 | 3.35 | 5.19 | 2.17 | 3.79 |
| 1.1 | 261 | 3.72 | 5.60 | 135 | 3.69 | 5.38 | 2.36 | 4.20 | 126 | 3.76 | 5.84 | 2.47 | 4.32 |
| 1.2 | 122 | 4.04 | 6.44 | 68 | 4.12 | 6.64 | 2.65 | 4.73 | 54 | 3.95 | 6.19 | 2.82 | 4.92 |
| 1.3 | 44 | 4.60 | 6.81 | 26 | 4.78 | 7.32 | 2.99 | 5.32 | 18 | 4.35 | 5.98 | 3.21 | 5.61 |
| 1.4 | 13 | 4.67 | 6.58 | 7 | 4.94 | 7.52 | 3.36 | 5.98 | 6 | 4.38 | 5.31 | 3.66 | 6.40 |
| 1.5 | 10 | 5.29 | 8.23 | 6 | 4.97 | 7.92 | 3.78 | 6.73 | 4 | 5.81 | 8.60 | 4.17 | 7.29 |

^*^ Compared by 2007 WHO/ICCIDD recommend reference

References:

[1] L. C. Foo, A. Zulfiqar, M. Nafikudin, M. T. Fadzil, and A. S. Asmah, "Local versus WHO/International Council for Control of Iodine Deficiency Disorders-recommended thyroid volume reference in the assessment of iodine deficiency disorders," *Eur J Endocrinol,* vol. 140, no. 6, pp. 491-7, 1999.

[2] F. Xu, K. Sullivan, R. Houston, J. Zhao, W. May, and G. Maberly, "Thyroid volumes in US and Bangladeshi schoolchildren: comparison with European schoolchildren," *Eur J Endocrinol,* vol. 140, no. 6, pp. 498-504, 1999.

[3] F. Azizi, H. Delshad, and Y. Mehrabi, "Thyroid volumes in schoolchildren of Tehran: comparison with European schoolchildren," *J Endocrinol Invest,* vol. 24, no. 10, pp. 756-62, 2001.

[4] A. Rossi, E. Tomimori, R. Camargo, and G. Medeiros-Neto, "Determination of thyroid volume by Sonography in healthy Brazilian schoolchildren," *J Clin Ultrasound,* vol. 30, no. 4, pp. 226-31, 2002.

[5] S. Darcan, P. Unak, O. Yalman, F. Y. Lambrecht, F. Z. Biber, D. Goksen, and M. Coker, "Determination of iodine concentration in urine by isotope dilution analysis and thyroid volume of school children in the west coast of Turkey after mandatory salt iodization," *Clin Endocrinol (Oxf),* vol. 63, no. 5, pp. 543-8, 2005.

[6] Y. Fuse, N. Saito, T. Tsuchiya, Y. Shishiba, and M. Irie, "Smaller thyroid gland volume with high urinary iodine excretion in Japanese schoolchildren: normative reference values in an iodine-sufficient area and comparison with the WHO/ICCIDD reference," *Thyroid,* vol. 17, no. 2, pp. 145-55, 2007.

[7] Z. Szybinski, M. Trofimiuk-Muldner, M. Buziak-Bereza, L. Walczycka, and A. Hubalewska-Dydejczyk, "Reference values for thyroid volume established by ultrasound in Polish schoolchildren," *Endokrynol Pol,* vol. 63, no. 2, pp. 104-9, 2012.

[8] D. Bonofiglio, S. Catalano, A. Perri, M. P. Baldini, S. Marsico, A. Tagarelli, D. Conforti, R. Guido, and S. Ando, "Beneficial effects of iodized salt prophylaxis on thyroid volume in an iodine deficient area of southern Italy," *Clin Endocrinol (Oxf),* vol. 71, no. 1, pp. 124-9, 2009.

[9] WHO/UNICEF/ICCIDD, *Assessment of iodine deficiency disorders and monitoring their elimination: A guide for program managers,* WHO, Geneva, 2007.
